# Socio-economic and cultural determinants of mothers and fathers for low birth weight newborns in the region of Marrakech (Morocco): A case-control study

**DOI:** 10.1371/journal.pone.0269832

**Published:** 2022-06-14

**Authors:** Mohamed Elaabsi, Mohamed Loukid, Saloua Lamtali

**Affiliations:** 1 Pharmacology Laboratory, Neurology, Anthropobiology and Environment, Faculty of Sciences Semlalia, University Cadi Ayyad Marrakech, Marrakech, Morocco; 2 Higher Institute of Nursing Professions and Health Techniques, Marrakech, Morocco; Flinders University, AUSTRALIA

## Abstract

**Background:**

Low birth weight (LBW) is defined as a birth weight less than 2500 g. It is an important predictor of early neonatal mortality, morbidity, and long-term health outcomes. The aim of this study was to identify risk factors for low birth weight in Marrakech Morocco.

**Methods:**

A retrospective based case-control study was conducted from July 2018 to July 2019. 462 mother infant pairs (231 low birth weight babies as cases and 231 normal birth weights as controls) were included in the study. Data were collected through face to face interview using a structured and pretested questionnaire. The collected data were managed with Statistical Package for Social Science (SPSS) version 20. Bivariate and multivariate binary logistic regression were used to identify factors associated with low birth weight at p-value < 0.05 with their respective odds ratios and 95% confidence interval.

**Results:**

The univariate analysis revealed the effect of the following determinants on the LBW: rural residence, father’s age, father’s professional activity, consanguinity, family type, mother’s low educational level, and mother’s intense physical activity. After the multivariate analysis, the risk factors identified were: rural residence (P = 0.017), father’s professional activity (temporarily working) (P = 0.000), absence of the consanguinity link (P = 0.016), and mother’s intense physical activity (P = 0.014).

**Conclusion:**

Results show father’s professional activity (temporarily working), rural residence, absence of the consanguinity link and mother’s intense physical activity are independent predictors of low birth weight. The current findings add substantially to the growing literature on the influence of parent’s socio-demographic and cultural factors on LBW in resource-constrained settings and provide empirical data for public health interventions to reduce low birth weight.

## Introduction

Low birth weight (LBW), defined as a birth weight less than 2500g, is a significant risk of death and disability [1]. Worldwide, 2.6 million newborns died in 2016 and half of them occurred in sub-Saharan Africa [2]. The most common causes of these deaths were birth asphyxia, infection, complications of preterm birth and birth defects in the early neonatal period [2]. Thirty percent of deaths were attributed to premature birth and low birth weight [3].

Globally, an estimated 15% to 20% of all births are LBW [4, 5]. The prevalence of low birth weight in Senegal, Burkina Faso, Malawi, Ghana, and Uganda was, respectively, 15.7%, 13.4%, 12.1%, 10.2%, and 10% [6]. In Morocco, fifteen percent of newborns have low birth weight [7]. In Algeria and Tunisia the percentages of LBW don’t exceed 7% [7].

In fact, the goal of the organization is that by the year 2025 the number of children born weighing less than 2500 g will have been reduced by 30% [8], which corresponds to a decrease of about 3% per year between 2012 and 2025.

In developing countries, the low birth weight rate is twice as high than in developed countries with 16.5% against 7%. Despite progress in socio-economic and health care in recent decades, the proportion of low birth weight infants is still high, and is even increasing in some countries [9].

A systematic review of low birth weight in Africa showed an increased risk of death, growth retardation and delayed neurodevelopment among very low birth weight and extremely low birth weight children [10].

Additionally, low birth weight is associated with a risk of hypertension later in life [11]. Many determinants of LBW were reported in studies. One cohort study showed that vomiting during the early trimester of pregnancy was associated with a higher risk of low birth weight [12]. In developing countries, maternal age, illiteracy, antenatal care follow-up, body mass index and socioeconomic status were predictors of low birth weight [13]. Antepartum hemorrhage, hypertensive disorders of pregnancy and primiparity were associated with low birth weight in a Gambian study [14]. Additionally, low birth weight was associated with maternal anemia [15] and malaria during pregnancy [16], intrauterine growth restriction and premature birth [17].

In other studies, educational level and occupational status [18], maternal stature and weight [19], hypertensive disorders during pregnancy [20], congenital malformations [21] and indoor pollution from the type of fuel used for cooking were, also, predictors of low birth weight.

On the other hand, multiple micronutrient supplements and preventive antimalarial drugs during pregnancy had a positive effect on low birth weight [4, 22]. Identifying risk factors for low birth weight in the Moroccan context have many benefits to set targeted preventive and treatment strategies. The findings of this study will add evidence for decision markers to establish appropriate management protocols, targeted to the study area. The study area is located in a region where neonatal mortality is high in the country [23]. Therefore, this study aimed to identify risk factors for low birth weight in hospitals and birthing center of the Marrakech region, Morocco.

## Materials and methods

### Study setting and study design

A retrospective case-control study was conducted from July 2018 to July 2019, at the maternity hospital of Ibn Zohr Hospital, Mother and Child Hospital CHU Mohammed VI; and at three health centers with a delivery module: Loudaya; Massera and Syba in Marrakech. These maternities recorded a very high number of deliveries. According to the statistics provided by the Health Delegation of the Marrakech-Safi Region, the total number of live newborns in 2017, at the CHU, was 14932 (Health Delegation of the Marrakech-Safi Region, 2017). Ibn Zohr Hospital and CHU Mohammed VI ensure 83% of deliveries in this region. The health services offered by these two establishments include prenatal consultations and postnatal monitoring of newborns.

The region of Marrakech-Safi covers an area of 41,404 km2 or 6% of the national territory and has 4520569 inhabitants (General Population and Housing Census, 2018). The density of 109 inhabitants per km^2^. The region includes 215 municipalities-divided into 18 urban and 197 rural. The capital of the region is the province of Marrakech.

### Study population

Newborns who were born in the two public hospitals and three health centers during the study period (12 months) were included in this study. Live newborns delivered at term without known risk factors (i.e. intrauterine growth restriction) of low birth weight were included in the study. Mothers with premature delivery (before 37 completed weeks of gestation) and mothers with medical status that would affect birth weight (i.e. hypertensive disorders of pregnancy, diabetes mellitus), were excluded from the study. Mothers who gave birth to neonates weighing less than 2500 grams were cases and neonates’ ≥2500 grams were controls.

### Study variables

The outcome/dependent variable was low birth weight. The exposure/independent variables were socio-economic and cultural variables (maternal and paternal age, education, occupation, residence, marital status, relationship, using tobacco, alcoholic, social and medical coverage and maternal physical activity (High physical activity: housework tasks or work outside).

### Sample size determination and sampling technique

The Sample size was calculated using the STATCALC program of EPI6, for unmatched case control with 95% confidence and 80% power to detect a minimum odds ratio of 2.0 assuming that the least prevalent factor will be present in minimum 10% of the controls as reported by Anand13 in his study. The final calculated sample size was 231 cases and 231 controls.

Both cases and controls were recruited on an ongoing basis until the required sample size was fulfilled.

### Sampling and data collection procedure

The hospitals and the health centers where the study takes out were selected purposely. Cases (birth weight less than 2500 grams) were included in the study and two consecutive mothers in the controls (birth weight ≥2500 grams) were interviewed. Data for the study were extracted from birth registers containing information about maternal and newborn characteristics using a structured database. Data were collected using a pre-tested and structured questionnaire through a face-to-face interview. The questionnaire includes information about: Socio-economic and cultural characteristics of mothers, Socio-economic and cultural characteristics of father’s, and characteristics of birth. The questionnaire was validated and pre-tested on 5% of the sample size in Mother and Child Hospital CHU Mohammed VI. The data was collected during two day at each hospital and one day at each health centers. Supervisors checked the completeness of the data.

### Statistical analysis

The statistical package for social sciences (SPSS) version 20.0-computer software was used for statistical analysis. Frequency distributions and cross tabulation between cases and controls were completed. Univariable and multivariable logistic regression analyses were computed in order to understand the effect of independent variables on the outcome variable.

The variables with p-value ≤0.2 in the univariate analysis were introduced into multivariable logistic regression analysis. Backward stepwise logistic regression method was used the Hosmer-Lemeshow test was used to assess goodness-of-fit. We considered p-value < 0.05 as level of significance.

### Ethical considerations

Official authorizations were obtained from the Regional Delegation of the Ministry of Health in Marrakech and from the Directorate of the Hospitals to access the maternity services and conduct this study. Informed verbal consent was obtained from study participants after being made informed of the objectives of the study. Confidentiality was guaranteed by keeping the anonymity of the respondents.

## Results

### Birth weight characteristics

The weight of all newborns ranges from 700g to 5000g and the mean is 2594.94g (standard deviation (SD) = 939.27g).

The boy/girl sex ratio was 1.22.

Table 1 shows some parameters of the birth weight of newborns for both samples.

**Table 1:** Characteristics of the birth weight distribution of the two samples.

	Cases	Controls
	N = 231	N = 231
**Minimum weight (g)**	700	2500
**Maximum weight (g)**	2400	5000
**Average weight (g)**	1806.86 ±ET	3383.01±ET
**Standard deviation**	529.42	490.38

The mean weight of newborns is 1806.86g (SD = 529.42) in LBW cases, and 3383.01g (SD = 490.38) in Control cases.

### Socio-economic and cultural characteristics

Table 2 shows the results of the univariate analysis of selected maternal and paternal characteristics with low birth weight. Mother physical activity was a significant factor of low birth weight (p = 0.000). Mothers in the case group were more active than those in the control group, successively 74.5% and 57.6%. Father’s age was also a significant factor of LBW (p = 0.011) with more young fathers among cases. About the parents’ place of residence, the cases were more likely from rural areas than controls, successively 53.2% and 35.1%. The difference was statistically significant (p = 0.000). The parents of controls babies had better social and medical coverage than the parents of LBW (44.2% and 41.1%). The mother’s education level was a determinant factor of LBW with 35.1% of illiteracy among cases versus 16% in controls (p = 0.000). Almost all the mothers were living with a partner, however a small difference between cases and controls was observed.

**Table 2:** Association of sociodemographic characteristics with low birth weight.

Characteristics	Cases	Controls	Univariate analysis
	N = 231(%)	N = 231(%)	Chi-square (X^2^)
**Residence**			**0.000** *
Rural	123 (53.2%)	81 (35.1%)	
Urban	94 (40.7%)	132 (57.1%)	
Suburban	14 (6.1%)	18 (7.8%)	
**Father’s age (years)**			**0.011** *
<= 30	108 (46.8%)	81 (35.1%)	
>30	123 (53.2%)	150 (64.9%)	
**Father’s level of education**			**0.287**
Illiterate	31 (13.4%)	31 (13.4%)	
Primary	121 (52.4%)	105 (45.5%)	
Secondary	73 (31.6%)	83 (35.9%)	
University	6 (2.6%)	12 (5.2%)	
**Father’s professional activity**			**0.000** *
Active	141 (61%)	215 (93.1%)	
Inactive	90 (39%)	16 (6.9%)	
**Consanguinity**			**0.000** *
Yes	20 (16.1%)	48 (20.8%)	
No	211 (91.3%)	183 (79.2%)	
**Father using tobacco**			**0.772**
Yes	54 (26.5%)	64 (27.7%)	
No	150 (73.5%)	167 (72.3%)	
**Father using alcohol**			**0.247**
Yes	7(3.3%)	13 (5.6%)	
No	203(96.7%)	218 (94.4%)	
**Family type**			**0.002** *
Extended family	136 (58.9%)	102 (44.2%)	
Nuclear family	95 (41.1%)	129 (55.8%)	
**Mother’s age (years)**			**0.223**
<= 20	34 (14.7%)	25 (10.8%)	
21–34	158 (68.4%)	155 (67.1%)	
≥ 35	39 (16.9%)	51 (22.1%)	
**Mother’s level of education**			**0.000** *
Illiterate	81 (35.1%)	37 (16%)	
Primary	81 (35.1%)	109 (47.2%)	
Secondary	64 (27.7%)	70 (30.3%)	
University	5 (2.2%)	15 (6.5%)	
**Mother’s professional activity**			**0.360**
Work	4 (1.7%)	7 (3%)	
Housewife	227 (98.3%)	224 (97%)	
**Mother’s marital status**			**0.408**
Living with a partner	226 (98.3%)	229 (99.1%)	
Living alone	4 (1.7%)	2 (0.9%)	
**Mother’s physical activity**			**0.000** *
High physical activity	172 (74.5%)	133 (57.6%)	
Low physical activity	59 (25.5%)	98 (42.4%)	
**Social and medical coverage**			**0.510**
Yes	95 (41.1%)	102 (44.2%)	
No	136 (58.9%)	129 (55.8%)	
**Mother using tobacco**			**0.369**
Yes	0 (0%)	2 (0.9%)	
No	229 (100%)	229 (99.1%)	
**Mother using alcohol**			**0.369**
Yes	0 (0%)	2 (0.9%)	
No	229 (100%)	229 (99.1%)	

*Significance Chi-square (X^2^) < 0.2.

A higher proportion of cases belonged to extended families (58.9%) than controls (44.2%). The difference was statistically significant (p = 0.002).

We identified in a first step the factors associated with LBW, then we performed a multivariate logistic modeling (Table 3). The variables kept for the model were: residence, father’s age, father’s professional activity, consanguinity, family type, mother’s education level and mother’s physical activity. We found that mother’s physical activity and consanguinity were associated with LBW. Area of residence and father’s professional activity were also associated with LBW, while mother’s level of education, family type and father’s age were not associated.

**Table 3:** Association between maternal and paternal socio-economic and cultural characteristics in our sample.

Characteristics	Univariate analysis (Chi-square (X^2^))	Multivariate analysis	
Factors	P value	OR (95% CI)	P
**Residence**	0.000	0.642 (0.446–0.923)	0.017*
**Father’s age (years)**	0.011	0.699 (0.456–1.073)	0.102
**Father’s Professional Activity**	0.000	1.980 (1.581–2.481)	0.000*
**Consanguinity**	0.000	2.088 (1.149–3.794)	0.016*
**Family type**	0.002	1.209 (0.793–1.845)	0.378
**Mother’s level of education**	0.000	0.775 (0.590–1.019)	0.068
**Mother’s physical activity**	0.000	0.578 (0.373–0.896)	0.014*

* Significance P< 0.05.

## Discussion

For the case, the weight of newborns varies from 700g to 2400g with an average of 1806.86g (SD = 529.42g), while for the controls, the weight of newborns varies from 2500g to 5000g with an average of 3383.01g (SD = 490.38g).

This calculated average value is lower than those found by Amor (1989) [24], Baali (1997) [25], Belkeziz (2000) [26] and Elkhoudri (2014) [27] for births in the city of Marrakech and which are respectively 3300g; 3350g; 3300g and 3277g. Nevertheless, there is a slight difference between the average of LBW of those studies and ours.

Socio-economic and cultural factors are determining factors in pregnancy monitoring. Their influence on pregnancy is related to the young age of the mother, ignorance of maternity problems, stress or excessive housework. The negative effect of the last two factors on pregnancy is known and widely described by authors [28].

In the present study, we did not find a significant correlation between maternal age and low birth weight. Similarly, Valero (2004) did not find any relationship between maternal age and fetal growth [29]. Afeke (2017), Maternal Age is often related to low birth weight, with a higher risk in the adolescence period and in women older than 35 years [30].

As for the age of the father, the majority of fathers in the study were over 30 years old, and logistic regression analysis revealed a significant connection (OR: 0.699; 95% CI: 0.456–1.073). Of all the studies reviewed, only two found that paternal age was also associated with a high probability of LBW [9, 31].

We also noticed that living in rural areas was significantly associated with LBW (OR: 0.642; 95% CI: 0.446–0.923). The same finding was reported by Abubakari (2015) in a study conducted in Ghana [32]. Other studies conducted in Ethiopia [33] and Bangladesh [34], reported an increased rate of LBW even in urban women.

In addition, the univariate analysis in this study highlighted the fact that low maternal education was strongly associated with LBW (p = 0.000). Without doubt, when mothers are highly educated, they attain improved nutritional status, health-seeking behaviors, and better maternal experiences with pregnancy and childcare. Furthermore, for those women, sexual initiation or increased contraceptive use to avoid pregnancy are obviously delayed [35, 36]. Thereby, the occurrence of LBW is reduced by avoiding early pregnancy.

The mother’s professional activity is not significantly associated with LBW (P = 0.360). Indeed, we found that the majority of mothers were jobless (housewives 98.3%). However, Al-dabbagh [37], noted that housewives have a significant risk of having a LBW newborn.

In this study, a higher proportion of cases had fathers who were unemployed or temporarily working (39%) than in the control group (6.9%), this difference was significantly related to LBW (OR: 1.980; 95% CI: 1.581–2.481).

Moreover, the absence of inbreeding between spouses are significantly connected with LBW (OR: 2.088; 95% CI: 1.149–3.794) similarly to the results found in many studies [38–40].

In the present study, a higher proportion of cases belonged to extended families (58.9%) than controls (44.2%). Similar to this, Vijayalaxmi in her study conducted in urban area of Bangalore found that majority of the women who delivered LBW babies were living in joint families (54.0%) [41].

Additionally, we noticed a positive association between high physical activity of the rural mother and LBW (OR: 0.578; 95% CI: 0.373–0.896). According to Laura [40], workers women have an increased risk having children with LBW. Camara [42], noted that the intense physical effort made by women in suburban areas in the fields in Senegal represented a risk factor for LBW.

What is more, active smoking is almost absent among the mothers in our study while passive smoking is not a determinant cause for low birth weight. Vahdaninia [43], noted it among the determinants of LBW, in contrast to Rodrigues [44], who demonstrated the absence of a significant association. Another reason why the rate of tobacco use is very low is that Moroccan women are generally attached to the moral values of the society.

## Conclusion

This study showed that Socio-economic and cultural characteristics were risk factors for low birth weight in the study areas. Residence, low professional activity of the fathers (temporarily working), consanguinity link and maternal physical activity were significantly associated with low birth weight. These findings contribute to the growing literature on the influence of maternal and paternal socio- economic and cultural factors on LBW in resource-constrained settings. Additionally, efforts should be done to improve living standard and lifestyles of mothers. Community based studies are needed to better address household and socio-economic factors with observation.

## Supporting information

S1 File (SAV)
